# Co-creating physical activity interventions: Findings from a multiple case study using mixed methods

**DOI:** 10.3389/fpubh.2022.975638

**Published:** 2022-09-21

**Authors:** Johanna Popp, Eva Grüne, Johannes Carl, Jana Semrau, Klaus Pfeifer

**Affiliations:** Department of Sport Science and Sport, Friedrich-Alexander-Universität Erlangen-Nürnberg, Erlangen, Germany

**Keywords:** co-production, participation, health promotion, cooperative planning, nursing care, automotive mechatronics, workplace, school

## Abstract

**Introduction:**

In health and physical activity promotion, there is growing interest in co-creation approaches that involve researchers and non-academic stakeholders in developing new interventions. Previous research has shown the promising results of cooperative planning as a co-creation approach in building new capacities and implementing physical activity-promoting interventions in nursing care and automotive mechatronics. However, it remains unclear whether (1) cooperative planning for physical activity promotion can be successfully transferred to other settings in the nursing care and automotive mechatronic sectors and (2) what key factors influence its success or failure.

**Methods:**

We conducted a multiple case study in three settings in the nursing care and automotive mechatronics sectors. Following a mixed methods approach, we collected, analyzed, and triangulated data from documents (*n* = 17), questionnaires (*n* = 66), and interviews (*n* = 6). Quantitative data were analyzed descriptively and through using nonparametric analyses of variance; qualitative data were analyzed using qualitative content analysis by extraction.

**Results:**

The transfer of cooperative planning to new settings was realized, though the impact varied by setting. While the interventions were developed and implemented in nursing care settings, interventions were developed but not implemented in the automotive mechatronics setting. In this context, intervention implementation was influenced by 11 key factors: *champion, commitment, embedment, empowerment, engagement, health-promoting leadership, ownership, relevance, resources, responsibility*, and *strategic planning*. Furthermore, the transfer of cooperative planning was influenced by different activity characteristics, namely *elaboration & reconsideration, group composition, number of meetings, participation, period, prioritization*, and *researchers' input & support*.

**Discussion:**

The present article contributes to a better understanding of a co-creation approach utilized for physical activity promotion and provides new insights into (1) the transferability of cooperative planning and (2) the associated key factors influencing intervention implementation. The success of cooperative planning varied by setting and was influenced by several activity characteristics and key factors, some of which showed complex relationships. This raises the question of whether some settings might benefit more from a co-creation approach than others. Therefore, future co-creation initiatives should carefully consider the specific characteristics of a setting to select and apply the most appropriate approach.

## Introduction

Synergizing the scientific world with the real world is considered a key benefit of co-creation (1). Indeed, co-creation approaches, in which researchers collaborate with non-academic stakeholders (i.e., end-users, practitioners, policy-makers) (2), are increasingly used to develop health-promoting interventions tailored to end-users and the given setting. On the one hand, tailoring interventions to end-users can increase their acceptability (3, 4) and effectiveness (5, 6). On the other hand, adapting interventions to the setting facilitates its contextualization by embedding these interventions into established routines and structures, utilizing existing resources, and building new capacities (7–9), in turn increasing the likelihood of sustained implementation (10–13).

In particular, the postulated fit of co-created interventions through the development of solutions that are suited to local circumstances makes this approach an appealing one for population groups that are characterized by specific needs and resources. Employees with higher levels of occupational physical activity (PA) are one such population group because PA is associated with fewer beneficial health effects for this group compared with employees with lower levels of occupational PA (14, 15). Following this, fostering the competencies needed to master physical demands in a healthy manner and adopt a physically active lifestyle might be a good focus of PA promotion for people with physically demanding occupations, rather than focusing solely on increasing PA levels (16).

Against this background, the research project Physical Activity-related Health Competence in Apprenticeship and Vocational Education (PArC-AVE), which was embedded in the research consortium Capital4Health, focused on PA promotion in the automotive mechatronics and nursing care sectors using a co-creation approach called cooperative planning (CP) (17, 18). CP engages non-academic stakeholders, including members of the target population, and researchers in an equal decision-making process to plan, develop, and implement interventions (19). Thus, CP exhibits parallels with other participatory or co-creation approaches (e.g., intervention mapping or community-based participatory research), but offers a unique constellation by combining the four key components of theory and goal orientation, involvement of all relevant stakeholders, knowledge co-production, and the use of progress monitoring and feedback loops (20). In the PArC-AVE project, the primary aim was to develop and implement new interventions to facilitate PA promotion within the given setting while taking the needs and resources of the end-users and setting into account. During the participatory development and implementation of the interventions involving end-users and other relevant actors from research, policy, and practice, the focus was on both the structural level by creating a PA-friendly environment and the individual level by promoting end-users' PA and physical activity-related health competence (PAHCO) (21, 22). Previous research examining CP in nursing care and automotive mechatronics has shown promising results when it comes to building new capacities and (sustainably) implementing PA-promoting interventions (23, 24).

Taking into account the concept of scaling up (25, 26) raises the question of the transferability of such approaches or interventions, i.e., the extent to which their impact could be achieved in another setting (27). More precisely, in our case, it remains unclear whether CP for PA promotion can be successfully transferred to other settings in the nursing care and automotive mechatronic sectors to reach and benefit more employees with physically demanding occupations. Additionally, the question arises as to what factors influence the success or failure of CP as a co-creation approach for PA promotion. The increasing number of studies using CP (28–31) or similar strategies in PA promotion and health promotion (11, 32, 33), along with the critical voices discussing the limitations and challenges of co-creation, such as the resources required or the risk of conflicts because of different interests (34, 35), underscore the need to explore these unanswered questions. Thus, the current study aims to address the following research questions:

1. How (un)successful is the transfer of CP for PA promotion to other settings in the nursing care and automotive mechatronic sectors? (transferability).
2. What key factors influence the success or failure of CP for PA promotion and, in particular, intervention implementation? (key factors).

## Methods

### Overview

**Figure 1:**
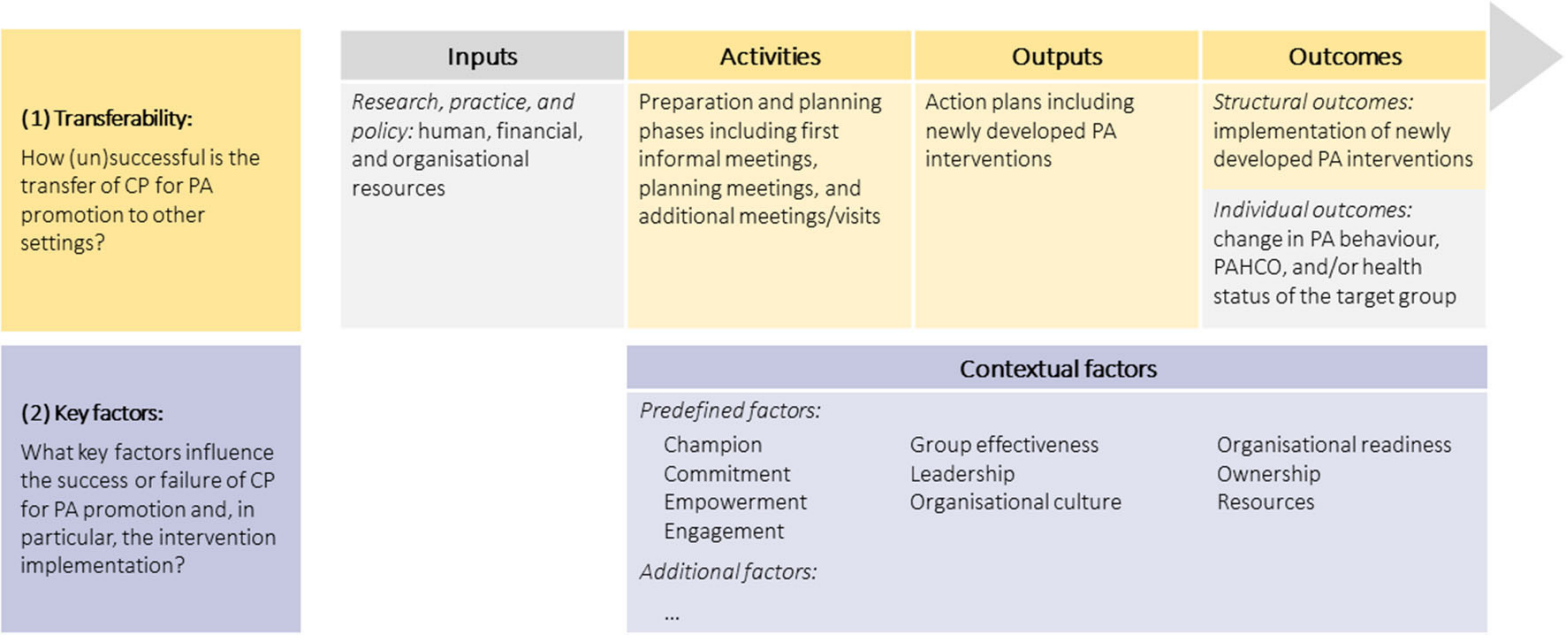
Logic model of the PArC-AVE project (including research questions). CP, cooperative planning; PA, physical activity; PAHCO, physical-activity related health competence.

To answer both research questions, we have used a multiple case study design with three different settings in the nursing care and automotive mechatronic sectors, with each representing one case. In each setting, a separate CP process was initiated in 2018 to develop and implement new multi-component interventions, each comprising multiple PA-promoting intervention components. These intervention components were expected to work best when implemented in combination but could also be implemented separately. The intervention implementation was not limited in time but was instead intended for the long term, if possible. The program activities, underlying evaluation theory, and planned methods have already been reported in detail in a study protocol (36). In brief, the evaluation of the transferability of CP and the key factors influencing its success or failure were based on a logic model illustrating the assumed mode of action of CP (see Figure 1). The logic model component *Activities* includes all project meetings and visits in the settings. *Outputs* are the direct products of the CP process, that is, the developed interventions documented in action plans, while *Outcomes* are the subsequent changes at the structural and individual levels. *Contextual factors* are defined as those factors influencing the CP process and its success or failure; these consist of factors that have been predefined based on previous project findings and a literature screening, as well as additional factors that have not yet been identified (36). Following the principles of a pragmatic evaluation (37, 38), we used a mixed methods approach to examine (1) the successful transfer of CP based on the *Activities, Outputs*, and *Structural outcomes* and (2) the key factors influencing the success or failure of CP, particularly the intervention implementation based on the *Contextual factors*. By comparing the results of all three settings, similarities and differences could be identified and aggregated to answer both research questions.

### Cases and participants

The multiple case study was undertaken in two state vocational education centers for health professions (Setting A: 200 nursing students enrolled in a nursing program, localized in a small city; Setting B: 180 nursing students enrolled in a nursing program, localized in a metropolis), and the assembly department of an automotive manufacturer (Setting C: 12,000 employees in the assembly department, localized in a large city), all located in Germany. The participants included end-users and other stakeholders involved in the CP processes. Table 1 provides more information about the final sample listed by the data sources.

**Table 1:** Description of the sample split by data sources.

**Data source**	**Research question *(logic model component)***	**Description of the sample**	
		**Number of documents**	
Structured minutes (qual)	Transferability *(Activities)*	**Total:** ***n*** **=** **14** A: *n* = 4 B: *n* = 4 C: *n* = 6	
Action plans (qual)	Transferability *(Outputs)*	**Total:** ***n*** **=** **3** A: *n* = 1 B: *n* = 1 C: *n* = 1	
		**Number of participants (participation rate)**	**Participants' characteristics**
ORIC questionnaires (quan)	Key factors *(Contextual factors)*	**Total:** ***n*** **=** **35** (94.6%) A: *n* = 16 (100%) B: *n* = 10 (83.3%) C: *n* = 9 (100%)	Not applicable *
CP questionnaires (quan)	Transferability and key factors *(Activities, Structural outcomes, Contextual factors*)	**Total:** ***n*** **=** **31** (54.4%) A: *n* = 8 (42.1%) B: *n* = 14 (77.8%) C: *n* = 9 (45%)	Role: A: 50% practitioners, 0% policy-makers, 50% end-users, 0% other B: 57.1% practitioners, 14.3% policy-makers, 28.6% end-users, 0% other C: 77.8% practitioners, 0% policy-makers, 11.1% end-users, 11.1% other
Interviews (qual)	Transferability and key factors *(Activities, Contextual factors)*	**Total:** ***n*** **=** **6** A: *n* = 2 B: *n* = 2 C: *n* = 2	Gender; working position: A: 100% female; head of the nursing education program, nursing teacher B: 100% female; head of the nursing school, nursing teacher C: 0% female; occupational physician, assembly department manager

* No information on participants' characteristics due to anonymous data collection.

A = Setting A; B = Setting B; C = Setting C; CP = cooperative planning; ORIC = organizational readiness for implementing change; qual = qualitative methods; quan = quantitative methods.

### Data collection

**Figure 2:**
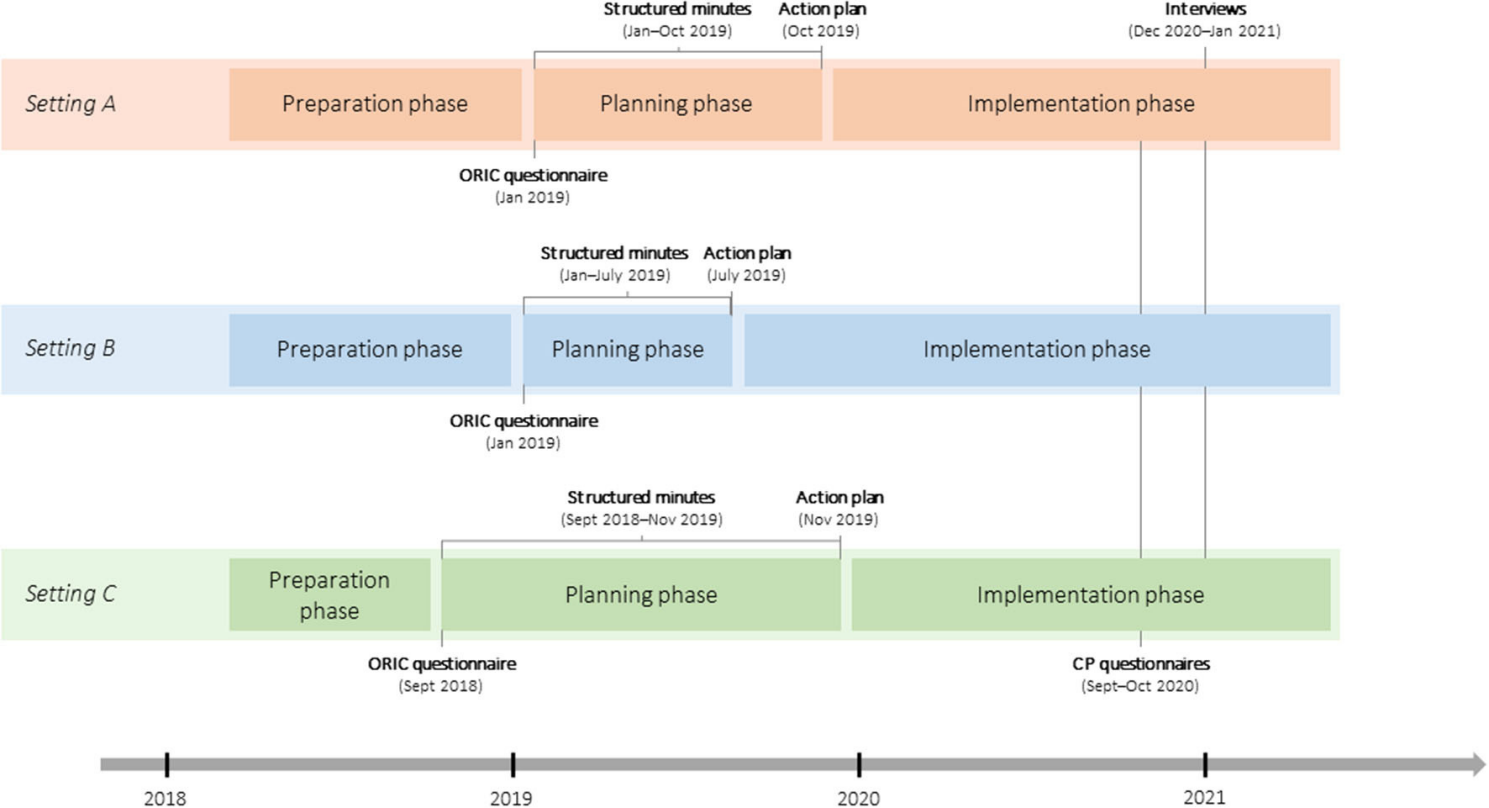
Cooperative planning processes and moments of data collections. CP, cooperative planning; ORIC, organizational readiness for implementing change.

Data were collected using quantitative and qualitative methods. To assess the transferability of CP based on planning meetings (*Activities*) and resulting multi-component interventions (*Outputs, Outcomes*), the data from structured minutes (qualitative), action plans (qualitative), questionnaires (quantitative), and interviews (qualitative) were used. Key factors influencing CP, particularly the intervention implementation (*Contextual factors*), were examined based on data from questionnaires (quantitative) and interviews (qualitative). The time points of measurement for all data sources are presented in Figure 2.

#### Quantitative data

In both questionnaire surveys, we used a maximum variation sampling technique to select the participants (39). To assess organizational readiness for change (40) as a predefined factor influencing CP, all stakeholders who attended the first planning meeting in each setting were invited to complete the Organizational Readiness for Implementing Change (ORIC) questionnaire (41), which had been translated into German [see study protocol (36)] at the beginning of the planning phase in September 2018 and January 2019. The questionnaire consisted of 12 items answered on a 5-point Likert scale (1 = disagree; 5 = agree). In this questionnaire survey, “change” refers to changes at the organizational level targeting PA promotion in the PArC-AVE project.

Furthermore, we utilized setting-specific CP questionnaires to evaluate the organization and realization of planning meetings, implementation status of intervention components, appraisal of the multi-component intervention, and predefined factors influencing CP. The development of these questionnaires is described in the study protocol (36); an overview of all items and subscales can be found in Supplementary material 2. The items were answered on a 5-point Likert scale (1 = disagree; 5 = agree). All stakeholders who attended at least one planning meeting in each setting were invited to complete the CP questionnaire from September to October 2020 in an online format using SoSci Survey ver. 3.2.12 (SoSci Survey GmbH, Munich, Germany).

#### Qualitative data

To collect detailed information on the planning meetings and number and characteristics of the involved actors, we took structured minutes of all planning meetings from September 2018 to November 2019. At the end of the planning phase, an action plan was created for each setting, documenting the number and description of the multi-component interventions developed (July–November 2019).

To identify the key factors influencing CP, particularly intervention implementation, we conducted semistructured interviews from December 2020 to January 2021. We developed setting-specific interview guides by building on data collected via structured minutes, action plans, and questionnaires (see Supplementary material 1). Following a purposeful sampling strategy of information-rich cases (39), we sought key informants with great knowledge about and influence on the PArC-AVE project. Accordingly, we selected two main stakeholders from each setting who were our contact persons and/or were substantially involved in the development and implementation of the interventions for the interviews. In Setting A, one invited stakeholder declined to participate because of a high workload, so another involved stakeholder representing a similar perspective was asked to participate. Two authors (EG and JP) conducted the interviews using the teleconferencing software Zoom Cloud Meetings (Zoom Video Communications, Inc., San Jose, USA). The interviews were audio-recorded and, on average, lasted about an hour (*SD* = 26.27; range 35.88–103.23 min).

### Data analyses

#### Quantitative data

Following the psychometric assessment studies by Shea et al. (41), we used the revised 10-item version of the ORIC questionnaire and analyzed mean scores of the 10-item total ORIC scale, the 5-item Change Commitment subscale, and the 5-item Change Efficacy subscale. The non-parametric Kruskal-Wallis test and Dunn-Bonferroni *post-hoc* tests were employed to examine differences across the settings. To compare the characteristics of the CP processes between the settings (i.e., planning meetings, implementation status, interventions' appraisal, influence of predefined factors), the CP questionnaire data were analyzed using the non-parametric Kruskal-Wallis test. Additionally, semantic differential charts were used to visualize the organization and realization of planning meetings and the influence of predefined factors across settings. The statistical analyses were performed using IBM SPSS Statistics ver. 26 (IBM, Armonk, USA); Microsoft Excel 2016 (Microsoft Corporation, Redmond, USA) with XLSTAT was used for the descriptive analysis. A significance level of *p* < 0.05 was applied for all analyses.

#### Qualitative data

The structured minutes and action plans were analyzed regarding the number and characteristics of planning meetings, involved actors, and intervention components using Microsoft Excel 2016 (Microsoft Corporation, Redmond, USA). The interviews were transcribed verbatim. Although analysis of the interview transcripts using qualitative content analysis according to Kuckartz (42) was initially planned in the study protocol (36), we decided to apply the qualitative content analysis procedure according to Gläser and Laudel (43, 44) instead. The main reason for this change was that Gläser and Laudel's content analysis focuses on the reconstruction of causal relationships, that is, between processes and outcomes, which is not supported by the coding procedure according to Kuckartz in this form. According to Gläser and Laudel (43, 44), the analysis starts with a set of theoretically derived categories, which is subsequently used for extracting relevant information from the interview transcripts. In our case, we referred to the logic model and our research questions to deductively define the categories of *activity characteristics influencing the transfer of CP* and *key factors influencing intervention implementation*. Then, two authors (EG and JP) developed the extraction rules, extracted the information from the text, and generated two extraction tables, one for each category. These tables include all information from the transcripts assigned to the respective categories. More precisely, the information was extracted in the following format: *subject* (one characteristic of the respective category labeled with a keyword), *content* (more detailed description of the subject), *reported cause* (information about a cause of the subject) and/or *reported effect* (information about the effect of the subject), and *source* (link to the relevant text passage in the transcript). These extraction tables were subsequently sorted by setting; the subjects were thematically grouped and summarized where appropriate and subsequently analyzed within and across settings. Microsoft Word 2016 (Microsoft Corporation, Redmond, USA) with MIA software (Ger.: Makrosammlung für qualitative Inhaltsanalyse; Eng.: macro collection for qualitative content analysis) (45) was used for the qualitative data analysis.

#### Data triangulation

Following the separate analyses, the quantitative and qualitative data were triangulated at the interpretation stage (46, 47) to provide a comprehensive description of transferability and key factors. First, the quantitative and qualitative findings were triangulated separately for each setting by identifying and comparing the main findings. Subsequently, patterns of similarity or difference were examined among the three settings. Two researchers (EG and JP) participated in the triangulation procedure to minimize potential bias in analyzing and interpreting the different findings. For discrepancies between researchers, consensus was reached through discussions.

## Results

To present the results split by the research questions, we built on the previously described and assigned logic model components of *Activities, Outputs, Structural outcomes*, and *Contextual factors*.

### Transferability: Success or failure of the transferred CP processes

#### Activities

Our analysis of structured minutes revealed differences in the number and time periods of meetings and number of involved actors among the three settings. The number of meetings varied from four in Settings A and B to six in Setting C, with each meeting lasting 3 h. The meetings took place over a period of 10 months in Setting A, 7 months in Setting B, and 14 months in Setting C. The number of involved actors varied between 17 and 19 in Setting A (*M* = 17.8), 7 and 15 in Setting B (*M* = 13.0), and 5 and 13 in Setting C (*M* = 10.5). The involved actors were researchers (Settings A, B, C: professor, research assistants) and non-academic stakeholders such as practitioners (Settings A, B: teachers, head of the nursing education program; Setting C: occupational physicians, occupational health referents, representative of the health insurance company, member of the works council, training center staff, assembly department manager), end-users (Settings A, B: nursing students; Setting C: assembly workers), and policy-makers (Settings A, B: headmasters, head of the nursing school; Setting C: none).

The analysis of the CP questionnaire data on the organization and realization of planning meetings yielded conspicuous findings. Across all settings, we found no significant differences for the items of the subscale *research*, namely, researchers' input, organization, guidance, and goal setting during CP. For example, the researchers' input revealed no significant differences across the settings (*H*(2) = 0.56, *p* = 0.755). However, for the other subscales *stakeholders, planning group*, and *benefits* of CP, significant differences between the settings were found for at least one item. For example, in terms of perceived *benefits*, significant differences across settings were identified for the perceived relevance of PA and health (*H*(2) = 11.86, *p* = 0.003), with higher scores for Setting A compared with Setting C. Details of the descriptive analysis and the significant differences for all subscales and across settings are presented in Supplementary material 2.

**Figure 3:**
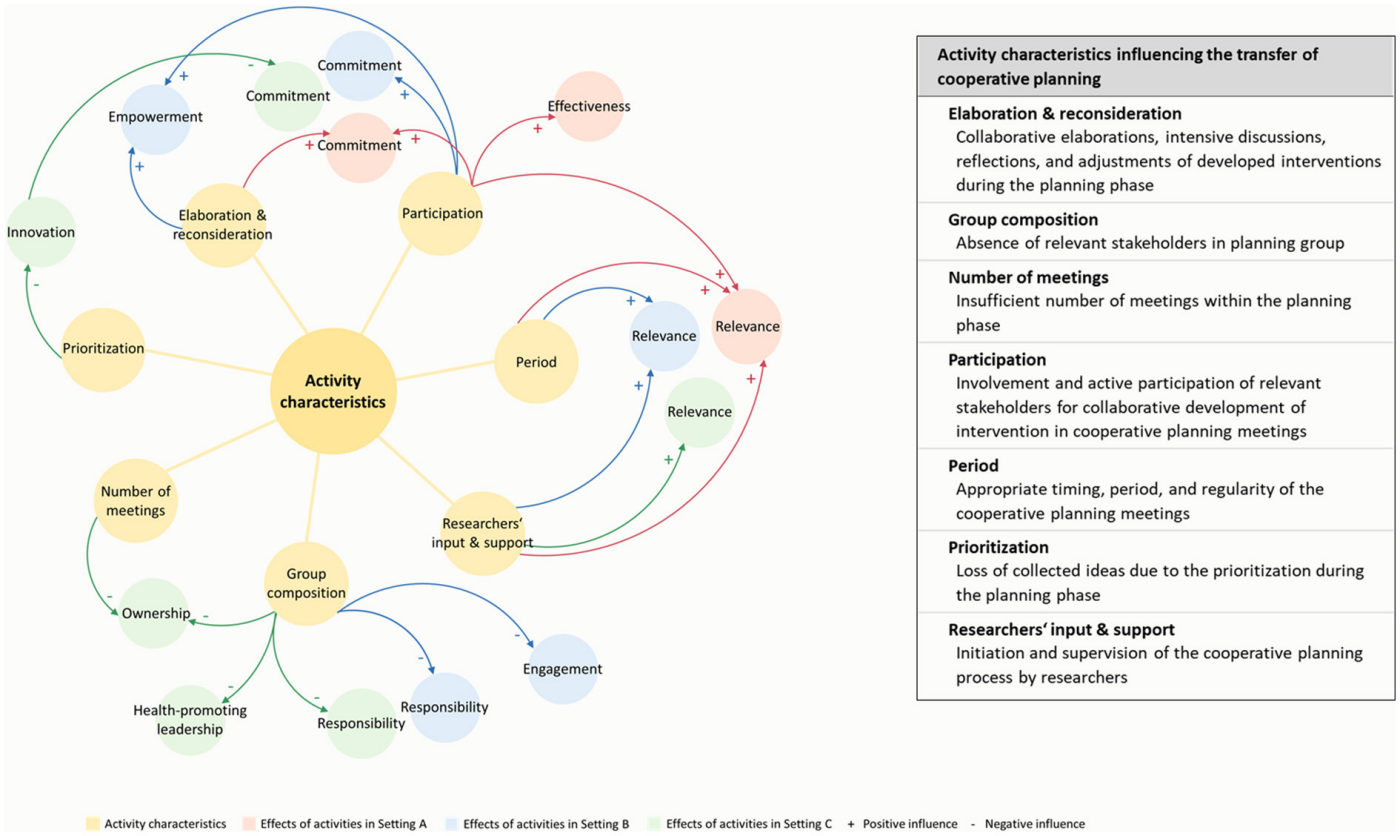
Activity characteristics influencing the transfer of cooperative planning identified through qualitative content analysis.

Qualitative content analysis of the interview data revealed the following seven subjects for the category *activity characteristics influencing the transfer of CP*: *elaboration & reconsideration, group composition, number of meetings, participation, period, prioritization*, and *researchers' input & support* (for detailed information, see Figure 3). In addition to the identified activity characteristics, we found numerous effects of these. For example, in all three settings, the identified activity characteristic *researchers' input & support* led to a high relevance of the project within the setting. In Settings A and B, the intensive *elaboration & reconsideration* during intervention development, the *participation* of relevant stakeholders (i.e., nursing students and teachers), and the *period* including timing and regularity of meetings had positive effects (e.g., the *elaboration & reconsideration* and *participation* positively influenced the empowerment of stakeholders to contribute to the intervention implementation). However, the absence of relevant stakeholders in the planning group (*group composition*) had negative effects in Settings B and C, such as a missing definition of responsibilities or low engagement of stakeholders to contribute to the intervention implementation. In Setting C, the insufficient *number of meetings* and *prioritization* of collected ideas during one meeting also had a negative effect. For example, prioritizing ideas led to a loss of innovation in interventions, in turn reducing commitment to the project and interventions. All discovered causal relationships between the identified activity characteristics and effects for the three settings are visualized in Figure 3.

#### Outputs and outcomes

According to the action plans, the planning meetings resulted in one newly developed multi-component intervention per setting, including 12 intervention components in Setting A, 11 in Setting B, and six in Setting C. Examples of the single components are the provision of information (intervention component *information for teachers* in Setting A), competence training (intervention component *training module PAHCO* in Setting C), or PA programs (intervention component *BuG lesson* in Settings A and B). An overview of the interventions, including a description of each intervention component, is provided in Supplementary material 3.

The analysis of the CP questionnaire data on the current implementation status and expected sustainability of the individual intervention components revealed heterogeneous results across the settings (see Supplementary material 3). Notably, only a few of the participants had information on the implementation status and sustainability of the intervention components; in addition, the participants' responses were not always consistent. Thus, the data analysis was based on an agreement rate of at least 66.7% (i.e., more than two thirds of the participants with information gave the same response) to make conclusive statements about the implementation status and expected sustainability. Overall, 33.3% of the intervention components (*n* = 4) were implemented in Setting A and 18.2% (*n* = 2) were implemented in Setting B. In Setting C, 33.3% of the intervention components (*n* = 2) were not perpetuated, and 16.7% (*n* = 1) were not implemented because of COVID-19 restrictions. Sustainable implementation of the intervention was rated as “possible” for 66.7% of the intervention components (*n* = 8) in Setting A, 63.6% (*n* = 7) in Setting B, and 0% (*n* = 0) in Setting C; it was rated as “not possible” for 8.3% of the intervention components (*n* = 1) in Setting A, 0% (*n* = 0) in Setting B, and 66.7% (*n* = 4) in Setting C. For a few intervention components, it was not possible to draw absolute conclusions regarding their implementation status or expected sustainability due to missing information from participants or inconclusive responses (i.e., agreement rate below 66.7%), leaving some percentages. The results of the appraisal of the intervention components regarding the creation of new capabilities, their effectiveness, their fit to the end-users and setting, and their perceived value within the organization can be found in Supplementary material 4.

### Key factors: Influence on the success or failures of CP

#### Contextual factors

Qualitative content analysis of the interview data revealed the following 11 different subjects for the category of *key factors influencing intervention implementation*: *champion, commitment, embedment, empowerment, engagement, health-promoting leadership, ownership, relevance, resources, responsibility*, and *strategic planning* (for more details, see Table 2). Eight of these key factors, that is, *commitment, embedment, engagement, health-promoting leadership, ownership, relevance, resources*, and *strategic planning*, were identified in all settings; the other three key factors were each found in two of the three settings. Whether these key factors had a positive or negative influence on intervention implementation depended on the reported availability or unavailability within the settings. For example, the availability of *commitment, engagement, health-promoting leadership, ownership*, and *strategic planning* in Settings A and B had a beneficial effect on intervention implementation, whereas the unavailability of these key factors hindered the intervention implementation in Setting C. Furthermore, the presence of a *champion* who is devoted to the project and manages it with enthusiasm facilitated intervention implementation in Setting A, whereas the non-presence of this very *champion* had a hindering effect on intervention implementation in Setting C; in Setting B, this key factor was not mentioned. Moreover, some key factors need to be considered in a more differentiated way because they both facilitated and hindered intervention implementation, such as *resources* in Settings A and C. For example, a lack of personal *resources* had a negative influence on intervention implementation, while the provision of financial *resources* had a beneficial effect. Examining the key factors with respect to intervention implementation in the different settings, successful intervention implementation was associated with a higher number of available key factors, with *n* = 10 key factors in Setting A, *n* = 9 in Setting B, and *n* = 2 in Setting C. Conversely, a high number of unavailable key factors were found in Setting C (*n* = 9), resulting in failed intervention implementation.

**Table 2:** Key factors influencing intervention implementation identified through qualitative content analysis.

**Key factors influencing intervention implementation**	**Setting A**	**Setting B**	**Setting C**
**Champion** Champion who is devoted to the project and manages it with enthusiasm	✓	n.m.	x
**Commitment** High degree of acceptance and advocacy of the project/intervention by stakeholders and end-users	✓	✓ / x	x
**Embedment** Embedment of the intervention in existing internal processes and structures	✓	✓	✓
**Empowerment** Development of abilities for autonomous intervention implementation by stakeholders	✓	✓	n.m.
**Engagement** High degree of engagement and willingness of stakeholders and end-users to contribute to the intervention implementation	✓	✓ / x	x
**Health-promoting leadership** Leadership support for the intervention implementation	✓	✓	x
**Ownership** Assumption of ownership of the project/intervention by the organization	✓	✓	x
**Relevance** High degree of relevance for PA promotion and high standing of the project	✓ / x	x	x
**Resources** Provision of financial, personnel, spatial-material and/or temporal resources for the intervention implementation	✓ / x	✓	✓ / x
**Responsibility** Definition and takeover of responsibilities for the intervention implementation	n.m.	✓	x
**Strategic planning** Execution of organizational and content-related planning of the intervention implementation	✓	✓	x

✓ = available; x = not available; n.m. = not mentioned.

**Figure 4:**
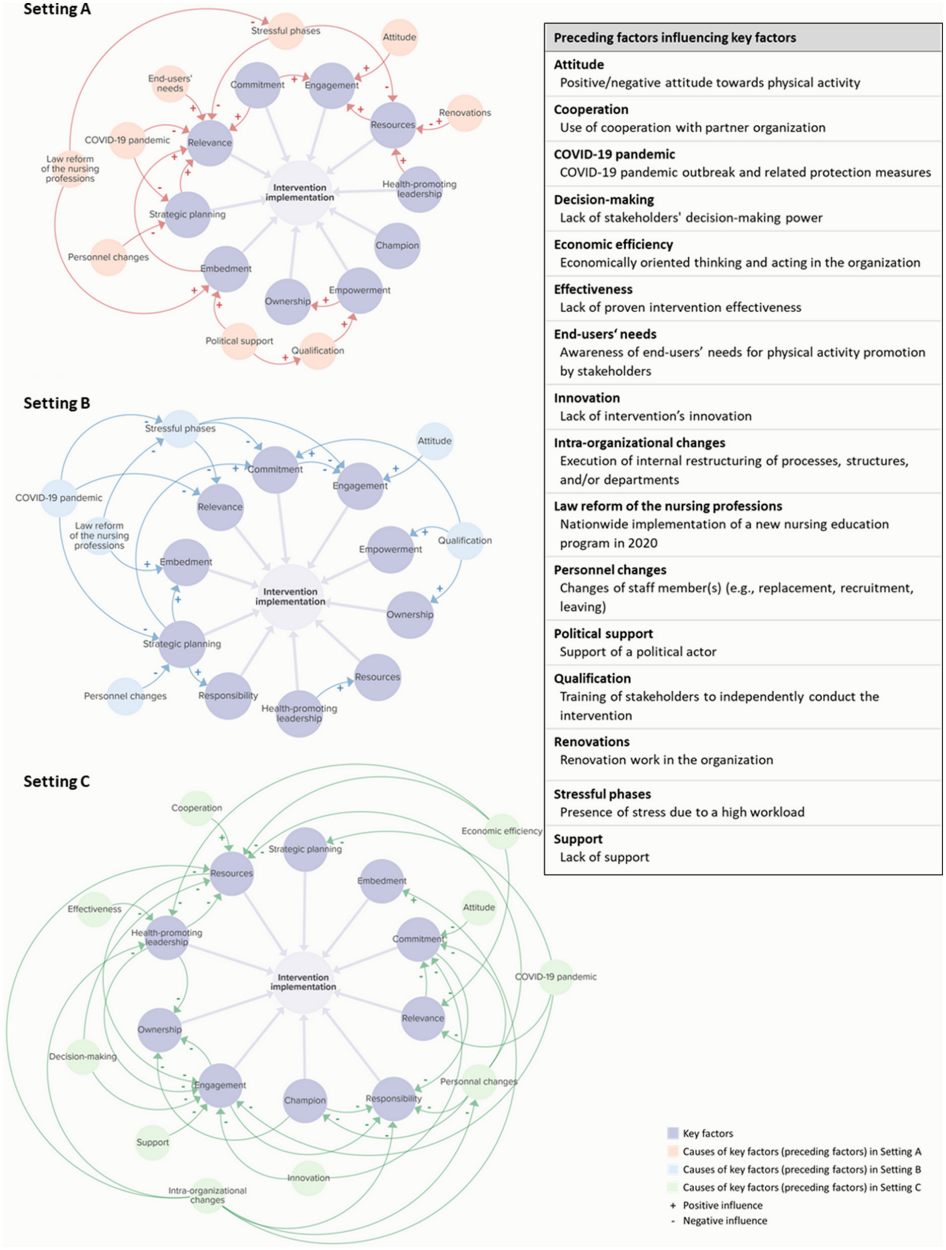
Reported causal relationships of key factors and preceding factors influencing intervention implementation identified through qualitative content analysis. The causal loop diagrams were produced using Kumu Inc (retrieved from https://kumu.io/).

During qualitative content analysis, we were able to extract not only the identified key factors, but also their reported effects and/or causes, thus establishing causal relationships. While the reported effects of the key factors were always associated with (un)successful intervention implementation, there were a variety of reported causes affecting the identified key factors. Hence, these causes behind the key factors can be referred to as the preceding factors. In contrast to the key factors, which showed high homogeneity across settings, the preceding factors were highly setting-specific. An example of a reported causal relationship between the preceding factor and key factor was the positive *attitude* toward PA, leading to high levels of *engagement* in Settings A and B, which, in turn, was a key factor facilitating intervention implementation. In Setting C, the lack of a positive *attitude* toward PA led to a low level of *commitment*, which emerged as a key factor that hindered intervention implementation. Furthermore, *COVID-19 pandemic* and *personnel changes* were found to be preceding factors in all three settings. Although these challenges were largely overcome through *strategic planning* in Settings A and B, they resulted in a missing *champion*, low *commitment*, low *engagement*, low *relevance*, lack of *responsibility*, and lack of *strategic planning* in Setting C. In addition to the identified causal relationships between the key factors and preceding factors, causal relationships were also revealed between the key factors themselves. For example, *health-promoting leadership* influenced the provision of *resources* in all three settings; while *health-promoting leadership* facilitated the provision of *resources* in Settings A and B, the provision of *resources* was deficient because of the lack of *health-promoting leadership* in Setting C. A detailed overview of all identified key factors and preceding factors, including their reported causal relationships, is illustrated in Figure 4.

The analysis of the CP questionnaire data on the predefined factors influencing CP yielded some significant differences across settings (see Supplementary material 2), underscoring the differences in the key factors identified in the interviews. For example, significant differences between settings were found for ownership Item 1 (*H*(2) = 10.37, *p* = 0.006) and empowerment Item 2 (*H*(2) = 7.08, *p* = 0.029), both with higher scores for Setting A compared with Setting C, while higher scores for Setting A compared with Setting B were found for engagement Item 2 (*H*(2) = 6.30, *p* = 0.043). As another predefined factor influencing CP, we also found differences in organizational readiness across settings. We observed significantly higher scores in total ORIC (*H*(2) = 7.83, *p* = 0.020) and Change Efficacy (*H*(2) = 9.00, *p* = 0.011) for Setting B compared with Setting C (see Supplementary material 5). No significant differences were found in the Change Commitment scores across all settings (*H*(2) = 4.61, *p* = 0.100).

## Discussion

### What is the key to successful intervention implementation?

The current study contributes to a better understanding of CP as a co-creation approach for promoting a physically active lifestyle by answering questions about (1) the transferability of CP and (2) the associated key factors influencing its success or failure, particularly intervention implementation. Overall, the transfer of CP to new settings in the nursing care and automotive mechatronic sectors was realized, though the achieved impact varied by setting. Comparing the results of the three settings, CP resulted in the development and implementation of intervention components in Settings A and B, whereas in Setting C, a multi-component intervention was developed but not implemented. In this context, 11 *key factors influencing intervention implementation* were identified: *champion, commitment, embedment, empowerment, engagement, health-promoting leadership, ownership, relevance, resources, responsibility*, and *strategic planning*. The identified key factors are confirmed by the implementation science literature in general (48, 49) and in the specific settings of schools (50, 51) and workplaces (52, 53). Moreover, these key factors show a high overlap with the contextual factors that we have predefined based on previous research (36).

It is striking that the key factors identified were very similar across the three settings, but the different manifestations of these factors seem to determine the implementation or non-implementation of interventions. Thus, the presence of numerous key factors in Settings A and B resulted in the implementation of interventions, whereas the absence of these factors led to the lack of intervention implementation in Setting C. In addition to the key factors, we identified preceding factors that had an impact on these very key factors and, thus, indirectly influenced intervention implementation. These preceding factors were characterized by a high degree of setting specificity. However, some of these factors are consistent with influencing factors reported in the implementation science literature, such as personnel changes, political support, and qualification in the school setting (50, 51) or intraorganizational changes, personnel changes, and support in the workplace setting (52, 53).

### The role of co-creation

By triangulating the quantitative and qualitative findings, we were able to uncover the relationships between activity characteristics and key factors. More precisely, some of the identified effects of activity characteristics corresponded to the identified key factors influencing intervention implementation. Thus, these activity characteristics seem to have had an impact on the manifestation of key factors, thereby also influencing intervention implementation.

In Setting A, for instance, all observed activity characteristics resulted in positive effects. For example, the *participation* of relevant stakeholders led to an increased *commitment* to and *relevance* of PA and health. In addition, both *commitment* and *relevance* were identified as key factors contributing to successful intervention implementation. The successful involvement of stakeholders was also supported by the results of the questionnaire survey, which showed high ratings for the subscale *stakeholders* in Setting A. The positive impact of stakeholder participation on commitment and relevance has also been the subject of other research articles (9, 12), indicating that partnerships between researchers and non-academic stakeholders are a promising approach for translating research findings into practice.

In comparison, in Setting B, not only the positive but also the negative effects of activity characteristics were found. For example, the *group composition* resulted in low *engagement* and a missing definition of *responsibilities*. These two effects of the *group composition* were also identified as key factors: *engagement* both facilitated and hindered intervention implementation, while *responsibility* facilitated intervention implementation. What might seem contradictory at first sight is a good example of the complexity of such processes and interactions of activity characteristics, key factors, and outcomes. For example, challenges can arise, while other factors simultaneously contribute to overcoming barriers (54), as the current study has uncovered in Setting B.

In Setting C, on the contrary, the observed activity characteristics mainly had negative effects. In this context, *group composition* appeared to be the most challenging, with negative effects on *health-promoting leadership, ownership*, and *responsibility*, all of which were identified as key factors and, thus, contributing to the failure of intervention implementation. The challenges associated with the *group composition* may have been caused by the lack of leadership participation, as well as the great heterogeneity of the involved practitioners (see the results of the structured minutes). More specifically, the lack of leadership participation may have hindered the decision-making process (see the preceding factor *decision making*, showing a lack of stakeholders' decision-making power). This is consistent with the findings from Nguyen et al. (55), emphasizing the importance of including decision- or policy-makers to achieve impact and implement the findings for integrated knowledge translation processes. Moreover, the great heterogeneity among practitioners may have increased the competing interests, which may have complicated the definition and adoption of responsibilities for intervention implementation. This illustrates a dilemma of co-creation because all relevant stakeholders should be involved (2), but at the same time, this increases the risk of conflicts arising from differing interests and perspectives (11, 35, 56).

Notably, the activity characteristic *researchers' input & support* had a positive effect on the relevance of the project in all three settings, underlining the importance of the researchers' role and contribution in the planning phase. This was supported by other studies highlighting the involvement of researchers as a key performance indicator for enhancing CP (19) and recommending that researchers work closely with end-users and other non-academic stakeholders from the outset of a co-creation process to ensure the relevance of findings (57). Overall, the current study highlights the complex and setting-specific interplay between activity characteristics and key factors, as well as the relevance of activity characteristics for the success or failure of the intervention implementation.

### Fit of co-creation approaches

The findings suggest that some settings might benefit more from a co-creation approach for PA promotion than others, with more favorable effects in the nursing care setting than in the automotive mechatronics setting. This may question a co-creation approach as a panacea leading to successful intervention implementation. Here, it might be advisable to consider in advance whether or, in particular, how the use of a co-creation approach is appropriate for a particular setting.

A first starting point to determine the fit of a co-creation approach can be the readiness for a change (40), such as PA promotion, in a specific setting. In the present study, we examined organizational readiness as a predefined factor influencing CP, here as operationalized by Shea et al. (41); our results failed to reveal that higher change efficacy and commitment comes with a more successful CP process. A recent review by Miake-Lye et al. (58) has shown that this organizational readiness assessment covers mainly the construct “readiness for implementation” as it is used in the Consolidated Framework for Implementation Research (CFIR; domain “inner setting”) (49). Concurrently, other organizational readiness for change assessments [e.g., (59, 60)] cover far more CFIR constructs (e.g., domains “characteristics of individuals,” “process”) (58). In this context, it may be important to consider more setting-specific information to classify a setting using a readiness scale to predict an organization's ability to conduct a change. However, implementation and especially determinant frameworks include relevant constructs and can be useful for mapping and developing a comprehensive organizational readiness instrument (58, 60, 61). For a more setting-specific application of the organizational readiness concept, the key factors of intervention implementation as identified in our multiple case study might also be useful for a readiness assessment. This readiness judgment should then be followed by a recommendation of strategies to enhance readiness before a co-creation process is conducted, for example, by identifying and preparing a champion [see the typology of readiness development strategies by Vax et al. (62)].

Second, classifying a setting as ready for change may not necessarily mean that this setting is also ready to engage in a co-creation process. Since participation is a core element of co-creation, a setting's readiness for participation, in which stakeholders' participation is considered important and valuable, is crucial for conducting a co-creation process. Vice versa, a setting completely closed to the stakeholders' participation may be unsuitable for a co-creation process (63). Moreover, participating in a co-creation process is not without costs for stakeholders because stakeholders' willingness and opportunities to invest additional resources are major requirements for conducting a co-creation process. Conversely, less emphasis may be placed on using a co-creation process when time or resources are limited (35). To determine a setting's readiness for participation, it might be useful to evaluate this readiness within the scope of an organizational readiness assessment, as done by Robertson et al. (64). This information can then be used to decide whether a co-creation approach seems suitable in a setting, prerequisites first need to be created (e.g., provision of resources), or another approach, such as implementing researcher-developed interventions, would be more appropriate.

Finally, a co-creation approach with the aim of PA promotion should be tailored to the unique needs and opportunities of the setting. This was supported by recent research emphasizing that co-creation is largely context-dependent (32, 57, 65), highlighting the need for localized solutions not only for the development of tailored interventions, but also for the realization of a co-creation process itself to account for the uniqueness of settings. Thus, a setting-specific selection of co-creation steps and principles or potential adaptations may be required to achieve an optimal fit between the chosen co-creation approach and given setting. In this regard, there is a growing body of literature focusing on providing guidance for the design of co-creation processes. For example, principles and strategies for partnerships with researchers and stakeholders (32, 66), or an instrument to help researchers select the appropriate tools to foster the impact of co-creation processes (67) are provided.

### Strengths and limitations

The current comprehensive mixed methods evaluation embedded in a multiple case study allowed us to gain new insights into the “black box” transferability and key factors of CP. Given the heterogeneity and flexibility of co-creation processes, this design was found to be appropriate for examining our research questions within and between three settings. In particular, the qualitative content analysis by extraction was a major strength because it enabled us not only to identify important activity characteristics and key factors, but also to determine the causal relationships between them and their reported causes and/or effects. This has given us a deep understanding of the dynamics and complexity of how these factors interact in the respective settings.

However, some limitations must be considered. First, as outlined in the study protocol, the measurement of outcomes at the individual level (i.e., PA behavior, PAHCO, health status) was planned in a pre-post design but finally not possible, as practitioners self-initiated the implementation of intervention components at an early stage (36). Therefore, in examining the transfer of CP, we refer to the logic model components *Activities, Outputs* and *Structural outcomes*. Second, the findings of the ORIC questionnaire should be interpreted with caution, as only a small sample size was reached, mainly because only people who participated in the first planning meeting took part in the survey. Third, we had a moderate response rate to the request for participation in the CP questionnaires; thus, not all the perspectives of the stakeholders on the organization and realization of planning meetings, the current implementation status and appraisal of interventions, and predefined factors influencing CP may be represented. However, we aimed to obtain missing information and gain deeper insights into the transferability and key factors of CP in different settings, here by conducting additional interviews and selecting interviewees through a purposeful sampling of information-rich cases. Fourth, the interview guide was pilot tested only within the research team, and the transcripts and findings were not returned to the interviewees for comments and feedback. Fifth, the identified causal relationships only refer to the interviewees' qualitative reports.

## Conclusion

The present article contributes to a better understanding of a co-creation approach utilized for PA promotion by providing new insights into (1) the transferability of CP as a co-creation approach and (2) the associated key factors influencing its success or failure, particularly intervention implementation. Specifically, the in-depth mixed methods evaluation in three settings in the nursing care and automotive mechatronic sectors provided relevant findings for future research. As a main result, transferring CP to new settings was achieved, though differences between the three settings were identified and demonstrated. Particularly, the achieved impact of CP varied by setting: while CP resulted in the development and implementation of PA-promoting interventions in nursing care settings, a multi-component intervention was developed but not implemented in the automotive mechatronics setting. In this context, we identified multiple key factors influencing intervention implementation and, thus, the success or failure of CP. These key factors also varied by setting, interacted in a complex way, and were related to co-creation activities. Therefore, future co-creation initiatives should carefully consider the specific characteristics of a setting to determine whether it is truly ready to initiate a change, such as PA promotion, and ready to engage in a co-creation process. Moreover, future research should investigate the complex and dynamic interactions between key factors to generate a theoretical foundation for the implementation and evaluation of such processes.

## Data availability statement

The raw data supporting the conclusions of this article will be made available by the authors, without undue reservation.

## Ethics statement

The studies involving human participants were reviewed and approved by Ethical Committee of the Friedrich-Alexander-Universität Erlangen-Nürnberg. The patients/participants provided their written informed consent to participate in this study.

## Author contributions

JP and EG conducted the qualitative and quantitative data collection, analyzed the qualitative and quantitative data, and drafted the manuscript. JC supported the qualitative and quantitative data collections and analyses. KP and JS acquired funding. KP supervised the work. All authors were involved in designing the study, interpreting data, critically reviewing drafts of the manuscript, and reading and approving the final manuscript.

## Funding

This research was conducted as part of the Physical Activity-related Health Competence in Apprenticeship and Vocational Education (PArC-AVE) project with the associated research network Capital4Health, which was funded by the German Federal Ministry of Education and Research (Grant No. 01EL1821A). The funder had no role in study design, data collection and analysis, decision to publish, and preparation of the manuscript.

## Conflict of interest

The authors declare that the research was conducted in the absence of any commercial or financial relationships that could be construed as a potential conflict of interest.

## Publisher's note

All claims expressed in this article are solely those of the authors and do not necessarily represent those of their affiliated organizations, or those of the publisher, the editors and the reviewers. Any product that may be evaluated in this article, or claim that may be made by its manufacturer, is not guaranteed or endorsed by the publisher.
