# Supplementary material for: Co-creating physical activity interventions: Findings from a multiple case study using mixed methods
Source: Front Public Health. 2022 Sep 21;10:975638. doi: 10.3389/fpubh.2022.975638 (PMC9534180; doi:10.3389/fpubh.2022.975638)
Supplement: Supplementary file 1 [file Table_1.docx]

Supplementary Material

# Additional File 1: Interview guide

Table 1: Interview guide for Setting A (original in German, a translated version is available on request from the corresponding authors)

| *Einführung:*  Es freut mich, dass Sie die Zeit gefunden haben, dieses Gespräch mit mir zu führen. Vor 2,5 Jahren hat das Projekt PArC-AVE an Ihrer Schule begonnen. Das Ziel war es, neue Maßnahmen zur Förderung der Bewegung und zur Stärkung der Bewegungsbezogenen Gesundheitskompetenz von PflegeschülerInnen an Ihrer Schule zu entwickeln.  Nun würde mich zunächst einmal interessieren – wie würden Sie denn Ihre Rolle im Projekt beschreiben? | | |
| --- | --- | --- |
| *Timeline:*  Wir beginnen heute mit einem Zeitstrahl, den Sie hier sehen. Auf diesem sind unsere gemeinsamen Treffen im Projekt PArC-AVE sowie weitere Projektereignisse abgebildet. Während der Planungstreffen haben wir gemeinsam neue Maßnahmen zur Bewegungsförderung entwickelt. Hier sehen Sie eine Liste der entwickelten Maßnahmen für Ihre Schule. In unserem Gespräch heute möchte ich mit Ihnen gerne einen Gesamtblick auf das Projekt werfen und unter anderem wichtige Ereignisse und Einflussfaktoren auf dem Zeitstrahl ergänzen. | | |
| **Inhaltliche Aspekte** | **Aufrechterhaltungsfragen** | **Konkrete Nachfragen** |
| *Leitfrage 1:*  Wenn Sie sich den Zeitstrahl nun in Ruhe ansehen… Was würden Sie sagen, welche weiteren wichtigen **Ereignisse oder Schlüsselmomente** sowohl innerhalb als auch außerhalb des Hauses haben das Projekt an Ihrer Schule beeinflusst? | | |
| Ergänzungen (intern, extern)  Corona | Sie haben … erwähnt. Können Sie das noch genauer beschreiben?  Haben Sie ein Beispiel dafür?  Woran machen Sie das fest?  Gibt es sonst noch etwas?  Wo würden Sie das auf dem Zeitstrahl einordnen? | Welche **Faktoren** haben dabei eine Rolle gespielt?  Welche Auswirkungen hatte denn die **Corona**-Pandemie? |
| *Leitfrage 2:*  Als nächstes würde mich interessieren, was denn dazu geführt hat, dass einige **Maßnahmen umgesetzt** worden sind, andere aber nicht? | | |
| Maßnahmenumsetzung  Organisation (Verantwortlichkeiten, Planungen)  Anpassungen | Sie haben … erwähnt. Können Sie das noch genauer beschreiben?  Haben Sie ein Beispiel dafür?  Woran machen Sie das fest?  Gibt es sonst noch etwas?  Wo würden Sie das auf dem Zeitstrahl einordnen?  Wann hat denn die **Umsetzung der ersten Maßnahmen** an Ihrer Schule begonnen? (Was hat dazu geführt, dass Maßnahmen bereits zu diesem Zeitpunkt umgesetzt wurden?) | Was denken Sie, wieso **ausgerechnet diese Maßnahmen** umgesetzt wurden?  Was **unterscheidet denn die Maßnahmen**, die umgesetzt wurden, von denen, die nicht umgesetzt wurden?  Was wäre denn notwendig, damit **auch die anderen Maßnahmen** umgesetzt werden?  Wie läuft das in Ihrem Haus so ab, wenn es um die Umsetzung der Maßnahmen geht? (Wie sehen denn die Planungen zur Umsetzung der Maßnahmen aus?)  Welche **Anpassungen** gab es, damit die Maßnahmen umgesetzt werden konnten? |
| *Leitfrage 3:*  Bei der Auswertung der Online-Umfrage haben wir festgestellt, dass die Bedeutung von Bewegungsförderung an Ihrer Schule sehr hoch eingeschätzt wird.  Gleichzeitig wurde angegeben, dass in hohem Maße **Verantwortung** für die entwickelten Maßnahmen übernommen werden konnte. Wieso, würden Sie sagen, ist dies gelungen? | | |
| Verantwortungsübernahme  Kümmerer (Support, Engagement)  Wissenschaftliche Betreuung  Stellenwert | Sie haben … erwähnt. Können Sie das noch genauer beschreiben?  Haben Sie ein Beispiel dafür?  Woran machen Sie das fest?  Gibt es sonst noch etwas?  Wo würden Sie das auf dem Zeitstrahl einordnen?  Ab wann, würden Sie sagen, hat Ihre Schule **Eigenverantwortung** für das Projekt übernommen? | Welche Rolle haben dabei **einzelne Personen** an Ihrer Schule gespielt?  Welche Rolle hat dabei die **Betreuung durch die WissenschaftlerInnen** der Universität Erlangen gespielt?  Welchen **Stellenwert** hat denn das Projekt im Vergleich zu anderen Aktivitäten an Ihrer Schule? (Inwiefern hat sich der Stellenwert des Projekts an Ihrer Schule verändert?) |
| *Leitfrage 4:*  Was denken Sie denn, inwiefern die **gemeinsame Entwicklung von Maßnahmen** innerhalb der Planungstreffen die Umsetzung der Maßnahmen beeinflusst hat? | | |
| Beurteilung Kooperative Planung  Alternativen  Einfluss Zufriedenheit | Sie haben … erwähnt. Können Sie das noch genauer beschreiben?  Haben Sie ein Beispiel dafür?  Woran machen Sie das fest?  Gibt es sonst noch etwas?  Wo würden Sie das auf dem Zeitstrahl einordnen? | Was, denken Sie denn, hätte anstatt der Planungstreffen **besser oder schlechter funktioniert**?  Wie **zufrieden** waren Sie denn mit den entwickelten Maßnahmen und inwiefern hat dies die Umsetzung der Maßnahmen beeinflusst? |
| *Leitfrage 5:*  Wenn Sie nun auf das Projekt zurückblicken, wie würden Sie dieses **im Gesamten** beurteilen? | | |
| Gesamteinschätzung Projekt  Erfolg/Misserfolg  Fazit | Sie haben … erwähnt. Können Sie das noch genauer beschreiben?  Haben Sie ein Beispiel dafür?  Woran machen Sie das fest?  Gibt es sonst noch etwas? | Woran machen Sie denn den **Erfolg/Misserfolg** des Projekts fest?  Wenn Sie noch einmal am Anfang des Projekts wären, was würden Sie **aus Ihrer heutigen Perspektive empfehlen**? (Und was würden Sie anders bzw. gleich machen?) |
| *Abschluss:*  Jetzt haben wir einiges besprochen. Gibt es denn aus Ihrer Sicht noch etwas, das bisher nicht zur Sprache gekommen ist, Ihnen aber wichtig ist? | | |
