# Supplementary material for: Co-creating physical activity interventions: Findings from a multiple case study using mixed methods
Source: Front Public Health. 2022 Sep 21;10:975638. doi: 10.3389/fpubh.2022.975638 (PMC9534180; doi:10.3389/fpubh.2022.975638)
Supplement: Supplementary file 3 [file Table_3.docx]

Supplementary Material

# Additional File 3: Description and implementation status of the multi-component interventions

Table 1: Description and implementation status of the multi-component intervention in Setting A (n = 8)

| **Intervention component and description** | **No information**  **(n)** | **Implementation status** | | | | | **Sustainability** | |
| --- | --- | --- | --- | --- | --- | --- | --- | --- |
|  |  | **Implemented**  **(n)** | **Planned**  **(n)** | **Not perpetuated**  **(n)** | **Not implemented**  **(n)** | **Not implemented due to COVID-19 restrictions**  **(n)** | **Sustainable implementation possible**  **(n)** | **Sustainable implementation not possible**  **(n)** |
| **BuG lesson**  Weekly 45-min lesson during regular school hours in each class, covering the theory and practice of PA and health | 0 | **7** | 1 | 0 | 0 | 0 | **8** | 0 |
| **Active breaks / active lessons**  Incorporation of PA into class time through adding short breaks or combining curriculum content with PA | 1 | 4 | 0 | 3 | 0 | 0 | **7** | 0 |
| **Regular PA time**  Regularly scheduled PA time in the form of different PA offers | 5 | 1 | 0 | 0 | 2 | 0 | **3** | 0 |
| **Healthy day for nursing students**  Initiation of a school-wide healthy day to impart knowledge and increase motivation in dealing with PA and health | 3 | 1 | 2 | 0 | 2 | 0 | 3 | 2 |
| **Information for nursing students**  Information about PA and health as well as the intervention developed within the PArC-AVE project | 5 | 2 | 0 | 0 | 0 | 1 | 2 | 1 |
| **Information for teachers**  Information about PA and health as well as the intervention developed within the PArC-AVE project | 6 | **2** | 0 | 0 | 0 | 0 | **2** | 0 |
| **Idea pool**  Collection and provision of ideas and materials for PA promotion | 5 | 2 | 1 | 0 | 0 | 0 | **3** | 0 |

| **PA as a topic for the teachers’ closed-conference**  Discussion of PA promotion at teachers' closed-conference | 6 | 0 | 0 | 1 | 1 | 0 | 0 | **2** |
| --- | --- | --- | --- | --- | --- | --- | --- | --- |
| **PAHCO in curriculum**  Integration of PAHCO into the school curriculum | 2 | **5** | 1 | 0 | 0 | 0 | **6** | 0 |
| **Extension of the kinesthetic lessons**  Extension of kinesthetic lessons with the aim of strengthening the nursing students’ competencies in dealing with occupational and physical stresses | 4 | 2 | 2 | 0 | 0 | 0 | **4** | 0 |
| **Extension of care planning**  Extension of care planning for care recipients by nursing students to include PA promotion | 4 | 2 | 0 | 1 | 0 | 1 | **3** | 1 |
| **Information for care institutions**  Information about the PArC-AVE project and developed intervention | 6 | **2** | 0 | 0 | 0 | 0 | 1 | 1 |
| **Sum of components with consistent agreement  (> 66.7%)** | | **4** | **-** | **-** | **-** | **-** | **8** | **1** |
| *BuG* Ger. ‘Bewegt und Gesund’, Eng. Physical activity and health; *PA* physical activity; *PAHCO* physical activity-related health competence; *PArC-AVE* physical activity-related health competence in vocational education and training; **bold** > 66.7% of the respondents with information agreed | | | | | | | | |

Table 2: Description and implementation status of the multi-component intervention in Setting B (n = 14)

| **Intervention component and description** | **No information**  **(n)** | **Implementation status** | | | | | **Sustainability** | |
| --- | --- | --- | --- | --- | --- | --- | --- | --- |
|  |  | **Implemented**  **(n)** | **Planned**  **(n)** | **Not perpetuated**  **(n)** | **Not implemented**  **(n)** | **Not implemented due to COVID-19 restrictions**  **(n)** | **Sustainable implementation possible**  **(n)** | **Sustainable implementation not possible**  **(n)** |
| **BuG lesson**  Weekly 45-min or 90-min lesson during regular school hours in each class, covering the theory and practice of PA and health | 1 | **9** | 3 | 0 | 1 | 0 | **12** | 1 |
| **Active breaks**  Incorporation of PA into class time through adding short breaks | 3 | **8** | 2 | 0 | 1 | 0 | **10** | 1 |
| **PA in breaks**  Provision of various sports equipment and facilities for nursing students during breaks | 2 | 1 | 5 | 1 | 5 | 0 | 7 | 5 |
| **BuG theme day**  Organization of a BuG theme day as an annual project in the first year of apprenticeship, offering nursing students the opportunity to develop and implement their own ideas on the topics of PA and health | 6 | 1 | 5 | 0 | 1 | 1 | **6** | 2 |
| **Information for nursing students**  Information about the PArC-AVE project and developed intervention | 7 | 2 | 3 | 1 | 1 | 0 | **5** | 2 |
| **Extension of the kinesthetic lessons**  Extension of kinesthetic lessons with the aim of developing competencies in dealing with occupational and physical stresses as well as in promoting PA in care recipients | 5 | 3 | 5 | 0 | 1 | 0 | **8** | 1 |
| **Back-strengthening work / working posture**  Integration of the aspect of back-strengthening work / working posture into the BuG lesson | 6 | 4 | 3 | 1 | 0 | 0 | **7** | 1 |

| **Motivation of care recipients**  Development of nursing students’ skills to motivate care recipients to be physically active through greater integration of counseling strategies, role-playing, and peer counseling into the curriculum | 6 | 4 | 2 | 0 | 1 | 1 | **7** | 1 |
| --- | --- | --- | --- | --- | --- | --- | --- | --- |
| **Information for practical trainers / head nurses**  Regular exchanges between stakeholders from school and practice, e.g., to raise practitioners' awareness of PA during breaks | 8 | 1 | 3 | 0 | 1 | 1 | 4 | 2 |
| **Cooperation with physiotherapy**  Strengthening the collaboration between nursing care and physiotherapy | 9 | 1 | 1 | 2 | 1 | 0 | 2 | 3 |
| **Bonus system**  Points system designed to incentivize participation in various physical activity promotion activities | 8 | 1 | 1 | 1 | 1 | 2 | 3 | 3 |
| **Sum of components with consistent agreement  (> 66.7%)** | | **2** | **-** | **-** | **-** | **-** | **7** | **-** |
| *BuG* Ger. ‘Bewegt und Gesund‘, Eng. Physical activity and health; *PA* physical activity; **bold** > 66.7% of the respondents with information agreed | | | | | | | | |

Table 3: Description and implementation status of the multi-component intervention in Setting C (n = 9)

| **Intervention component and description** | **No information**  **(n)** | **Implementation status** | | | | | **Sustainability** | |
| --- | --- | --- | --- | --- | --- | --- | --- | --- |
|  |  | **Implemented**  **(n)** | **Planned**  **(n)** | **Not perpetuated**  **(n)** | **Not implemented**  **(n)** | **Not implemented due to COVID-19 restrictions**  **(n)** | **Sustainable implementation possible**  **(n)** | **Sustainable implementation not possible**  **(n)** |
| **Training module PAHCO**  Imparting knowledge about PA and health and how to deal with occupational stress through training of employees at the training center and consultations at the workplace by trained trainers | 6 | 0 | 0 | 0 | 0 | **3** | 1 | 2 |
| **Tutoring system**  Employees participate voluntarily in a workshop enabling them to act as tutors to promote PA and health among their colleagues | 4 | 0 | 0 | **4** | 0 | 1 | 0 | **5** |
| **Information campaign for group leaders**  Information about PA and health as well as the intervention developed within the PArC-AVE project | 6 | 0 | 1 | 0 | 1 | 1 | 2 | 1 |
| **Opportunities for keeping fit**  Creating various opportunities for employees to become physically active | 5 | 0 | 0 | **3** | 0 | 1 | 0 | **4** |
| **Information strategy**  Information about PA and health as well as the intervention developed within the PArC-AVE project | 7 | 0 | 0 | 0 | 1 | 1 | 0 | **2** |
| **Coordinator / steering group**  Coordination and implementation of the developed intervention by (a) specific person(s) | 7 | 0 | 0 | 1 | 0 | 1 | 0 | **2** |
| **Sum of components with consistent agreement  (> 66.7%)** | | **-** | **-** | **2** | **-** | **1** | **-** | **4** |
| *PA* physical activity; *PAHCO* physical activity-related health competence; **bold** > 66.7% of the respondents with information agreed | | | | | | | | |
