# Supplementary material for: Co-creating physical activity interventions: Findings from a multiple case study using mixed methods
Source: Front Public Health. 2022 Sep 21;10:975638. doi: 10.3389/fpubh.2022.975638 (PMC9534180; doi:10.3389/fpubh.2022.975638)
Supplement: Supplementary file 4 [file Table_4.docx]

Supplementary Material

# Additional File 4: Appraisal of the multi-component interventions

Table 1: Appraisal of the intervention components in Setting A

| **The intervention component “BuG lesson (‘PA and health lesson’)“…** | | | | | | | | |
| --- | --- | --- | --- | --- | --- | --- | --- | --- |
| … offers the apprentices a new capability to be physically active. |  | Does not apply |  | Strongly applies | |  | | *n* = 8  *M* = 3.38 |
| … contributes to the apprentices getting more physically active. |  | Does not apply |  | Strongly applies | |  | | *n* = 8  *M* = 3.38 |
| … is tailored to the needs and requirements of the apprentices. |  | Does not apply |  | Strongly applies | |  | | *n* = 8  *M* = 2.75 |
| … fits to the situation and the organizational conditions in our organization. |  | Does not apply |  | Strongly applies | |  | | *n* = 8  *M* = 3.00 |
| … has a great value in our organization compared to other activities/goals. |  | Does not apply |  | Strongly applies | |  | | *n* = 8  *M* = 2.38 |
| **The intervention component “Active breaks / active lesson”** | | | | | | | | |
| … offers the apprentices a new capability to be physically active. |  | Does not apply |  | Strongly applies | |  | | *n* = 7  *M = 3.43* |
| … contributes to the apprentices getting more physically active. |  | Does not apply |  | Strongly applies | |  | | *n* = 7  *M* = 3.00 |
| … is tailored to the needs and requirements of the apprentices. |  | Does not apply |  | Strongly applies | |  | | *n* = 7  *M* = 2.57 |
| … fits to the situation and the organizational conditions in our organization. |  | Does not apply |  | Strongly applies | |  | | *n* = 7  *M* = 3.29 |
| … has a great value in our organization compared to other activities/goals. |  | Does not apply |  | Strongly applies | |  | | *n* = 7  *M* = 2.43 |
| **The intervention component “Regular physical activity times“…** | | | | | | | | |
| … offers the apprentices a new capability to be physically active. |  | Does not apply |  | Strongly applies | |  | | *n* = 3  *M* = 2.67 |
| … contributes to the apprentices getting more physically active. |  | Does not apply |  | Strongly applies | |  | | *n* = 3  *M* = 2.67 |
| … is tailored to the needs and requirements of the apprentices. |  | Does not apply |  | Strongly applies | |  | | *n* = 3  *M* = 2.67 |
| … fits to the situation and the organizational conditions in our organization. |  | Does not apply |  | Strongly applies | |  | | *n* = 3  *M* = 2.67 |
| … has a great value in our organization compared to other activities/goals. |  | Does not apply |  | Strongly applies | |  | | *n* = 3  *M* = 1.67 |
| **The intervention component “Health day for nursing students“…** | | | | | | | | |
| … offers the apprentices a new capability to be physically active. |  | Does not apply |  | Strongly applies | |  | | *n* = 5  *M* = 2.80 |
| … contributes to the apprentices getting more physically active. |  | Does not apply |  | Strongly applies | |  | | *n* = 5  *M* = 3.00 |
| … is tailored to the needs and requirements of the apprentices. |  | Does not apply |  | Strongly applies | |  | | *n* = 5  *M* = 2.80 |
| … fits to the situation and the organizational conditions in our organization. |  | Does not apply |  | Strongly applies | |  | | *n* = 5  *M* = 2.20 |
| … has a great value in our organization compared to other activities/goals. |  | Does not apply |  | Strongly applies | |  | | *n* = 5  *M* = 1.60 |
| **The intervention component “Information for nursing students“…** | | | | | | | | |
| … offers the apprentices a new capability to be physically active. |  | Does not apply |  | Strongly applies |  | | *n* = 3  *M* = 2.67 | |
| … contributes to the apprentices getting more physically active. |  | Does not apply |  | Strongly applies |  | | *n* = 3  *M* = 2.67 | |
| … is tailored to the needs and requirements of the apprentices. |  | Does not apply |  | Strongly applies |  | | *n* = 3  *M* = 2.67 | |
| … fits to the situation and the organizational conditions in our organization. |  | Does not apply |  | Strongly applies |  | | *n* = 3  *M* = 2.67 | |
| … has a great value in our organization compared to other activities/goals. |  | Does not apply |  | Strongly applies |  | | *n* = 3  *M* = 2.67 | |
| **The intervention component “Information for teachers“…** | | | | | | | | |
| … offers the apprentices a new capability to be physically active. |  | Does not apply |  | Strongly applies |  | | *n* = 2  *M* = 4.00 | |
| … contributes to the apprentices getting more physically active. |  | Does not apply |  | Strongly applies |  | | *n* = 2  *M* = 4.00 | |
| … is tailored to the needs and requirements of the apprentices. |  | Does not apply |  | Strongly applies |  | | *n* = 2  *M* = 4.00 | |
| … fits to the situation and the organizational conditions in our organization. |  | Does not apply |  | Strongly applies |  | | *n* = 2  *M* = 4.00 | |
| … has a great value in our organization compared to other activities/goals. |  | Does not apply |  | Strongly applies |  | | *n* = 2  *M* = 4.00 | |
| **The intervention component “Idea pool“…** | | | | | | | | |
| … offers the apprentices a new capability to be physically active. |  | Does not apply |  | Strongly applies | |  | | *n* = 3  *M* = 3.33 |
| … contributes to the apprentices getting more physically active. |  | Does not apply |  | Strongly applies | |  | | *n* = 3  *M* = 2.67 |
| … is tailored to the needs and requirements of the apprentices. |  | Does not apply |  | Strongly applies | |  | | *n* = 3  *M* = 2.67 |
| … fits to the situation and the organizational conditions in our organization. |  | Does not apply |  | Strongly applies | |  | | *n* = 3  *M* = 3.00 |
| … has a great value in our organization compared to other activities/goals. |  | Does not apply |  | Strongly applies | |  | | *n* = 3  *M* = 2.33 |
| **The intervention component “Physical activity as topic for the teachers’ closed-conference“…** | | | | | | | | |
| … offers the apprentices a new capability to be physically active. |  | Does not apply |  | Strongly applies |  | | *n* = 2  *M* = 4.00 | |
| … contributes to the apprentices getting more physically active. |  | Does not apply |  | Strongly applies |  | | *n* = 2  *M* = 4.00 | |
| … is tailored to the needs and requirements of the apprentices. |  | Does not apply |  | Strongly applies |  | | *n* = 2  *M* = 3.00 | |
| … fits to the situation and the organizational conditions in our organization. |  | Does not apply |  | Strongly applies |  | | *n* = 2  *M* = 2.50 | |
| … has a great value in our organization compared to other activities/goals. |  | Does not apply |  | Strongly applies |  | | *n* = 2  *M* = 1.00 | |
| **The intervention component “Physical Activity-related Health Competence in curriculum“…** | | | | | | | | |
| … offers the apprentices a new capability to be physically active. |  | Does not apply |  | Strongly applies |  | | *n* = 6  *M* = 3.17 | |
| … contributes to the apprentices getting more physically active. |  | Does not apply |  | Strongly applies |  | | *n* = 6  *M* = 3.00 | |
| … is tailored to the needs and requirements of the apprentices. |  | Does not apply |  | Strongly applies |  | | *n* = 6  *M* = 3.00 | |
| … fits to the situation and the organizational conditions in our organization. |  | Does not apply |  | Strongly applies |  | | *n* = 6  *M* = 3.00 | |
| … has a great value in our organization compared to other activities/goals. |  | Does not apply |  | Strongly applies |  | | *n* = 6  *M* = 2.67 | |
| **The intervention component “Extension of the kinesthetic lessons“…** | | | | | | | | |
| … offers the apprentices a new capability to be physically active. |  | Does not apply |  | Strongly applies | |  | | *n* = 4  *M* = 1.75 |
| … contributes to the apprentices getting more physically active. |  | Does not apply |  | Strongly applies | |  | | *n* = 4  *M* = 1.75 |
| … is tailored to the needs and requirements of the apprentices. |  | Does not apply |  | Strongly applies | |  | | *n* = 4  *M* = 2.00 |
| … fits to the situation and the organizational conditions in our organization. |  | Does not apply |  | Strongly applies | |  | | *n* = 4  *M* = 3.00 |
| … has a great value in our organization compared to other activities/goals. |  | Does not apply |  | Strongly applies | |  | | *n* = 4  *M* = 1.75 |
| **The intervention component “Extension of care planning“…** | | | | | | | | |
| … offers the apprentices a new capability to be physically active. |  | Does not apply |  | Strongly applies | |  | | *n* = 4  *M* = 1.75 |
| … contributes to the apprentices getting more physically active. |  | Does not apply |  | Strongly applies | |  | | *n* = 4  *M* = 1.75 |
| … is tailored to the needs and requirements of the apprentices. |  | Does not apply |  | Strongly applies | |  | | *n* = 4  *M* = 2.00 |
| … fits to the situation and the organizational conditions in our organization. |  | Does not apply |  | Strongly applies | |  | | *n* = 4  *M* = 3.00 |
| … has a great value in our organization compared to other activities/goals. |  | Does not apply |  | Strongly applies | |  | | *n* = 4  *M* = 1.75 |
| **The intervention component “Information for care institutions“…** | | | | | | | | |
| … offers the apprentices a new capability to be physically active. |  | Does not apply |  | Strongly applies | |  | | *n* = 2  *M* = 3.00 |
| … contributes to the apprentices getting more physically active. |  | Does not apply |  | Strongly applies | |  | | *n* = 2  *M* = 3.00 |
| … is tailored to the needs and requirements of the apprentices. |  | Does not apply |  | Strongly applies | |  | | *n* = 2  *M* = 3.00 |
| … fits to the situation and the organizational conditions in our organization. |  | Does not apply |  | Strongly applies | |  | | *n* = 2  *M* = 3.00 |
| … has a great value in our organization compared to other activities/goals. |  | Does not apply |  | Strongly applies | |  | | *n* = 2  *M* = 2.00 |

Table 2: Appraisal of the intervention components in Setting B

| **The intervention component “BuG lesson (Physical activity and health lesson)“…** | | | | | | |
| --- | --- | --- | --- | --- | --- | --- |
| … offers the apprentices a new capability to be physically active. |  | Does not apply |  | Strongly applies |  | *n* = 13  *M* = 3.31 |
| … contributes to the apprentices getting more physically active. |  | Does not apply |  | Strongly applies |  | *n* = 13  *M* = 3.15 |
| … is tailored to the needs and requirements of the apprentices. |  | Does not apply |  | Strongly applies |  | *n* = 13  *M* = 2.77 |
| … fits to the situation and the organizational conditions in our organization. |  | Does not apply |  | Strongly applies |  | *n* = 13  *M* = 2.92 |
| … has a great value in our organization compared to other activities/goals. |  | Does not apply |  | Strongly applies |  | *n* = 13  *M* = 2.38 |
| **The intervention component “Active breaks”** | | | | | | |
| … offers the apprentices a new capability to be physically active. |  | Does not apply |  | Strongly applies |  | *n* = 11  *M* = 3.09 |
| … contributes to the apprentices getting more physically active. |  | Does not apply |  | Strongly applies |  | *n* = 11  *M* = 2.64 |
| … is tailored to the needs and requirements of the apprentices. |  | Does not apply |  | Strongly applies |  | *n* = 11  *M* = 2.82 |
| … fits to the situation and the organizational conditions in our organization. |  | Does not apply |  | Strongly applies |  | *n* = 11  *M* = 2.91 |
| … has a great value in our organization compared to other activities/goals. |  | Does not apply |  | Strongly applies |  | *n* = 11  *M* = 2.55 |
| **The intervention component “Physical activity in breaks”** | | | | | | |
| … offers the apprentices a new capability to be physically active. |  | Does not apply |  | Strongly applies |  | *n* = 12  *M* = 2.42 |
| … contributes to the apprentices getting more physically active. |  | Does not apply |  | Strongly applies |  | *n* = 12  *M* = 2.50 |
| … is tailored to the needs and requirements of the apprentices. |  | Does not apply |  | Strongly applies |  | *n* = 12  *M* = 2.08 |
| … fits to the situation and the organizational conditions in our organization. |  | Does not apply |  | Strongly applies |  | *n* = 12  *M* = 1.92 |
| … has a great value in our organization compared to other activities/goals. |  | Does not apply |  | Strongly applies |  | *n* = 12  *M* = 1.92 |
| **The intervention component “'Physical activity and health' theme day“** | | | | | | |
| … offers the apprentices a new capability to be physically active. |  | Does not apply |  | Strongly applies |  | *n* = 8  *M* = 2.63 |
| … contributes to the apprentices getting more physically active. |  | Does not apply |  | Strongly applies |  | *n* = 8  *M* = 2.63 |
| … is tailored to the needs and requirements of the apprentices. |  | Does not apply |  | Strongly applies |  | *n* = 8  *M* = 2.63 |
| … fits to the situation and the organizational conditions in our organization. |  | Does not apply |  | Strongly applies |  | *n* = 8  M = 2.75 |
| … has a great value in our organization compared to other activities/goals. |  | Does not apply |  | Strongly applies |  | *n* = 8  *M* = 2.50 |
| **The intervention component “Information for nursing students”** | | | | | | |
| … offers the apprentices a new capability to be physically active. |  | Does not apply |  | Strongly applies |  | *n* = 7  *M* = 2.86 |
| … contributes to the apprentices getting more physically active. |  | Does not apply |  | Strongly applies |  | *n* = 7  *M* = 2.71 |
| … is tailored to the needs and requirements of the apprentices. |  | Does not apply |  | Strongly applies |  | *n* = 7  *M* = 2.57 |
| … fits to the situation and the organizational conditions in our organization. |  | Does not apply |  | Strongly applies |  | *n* = 7  *M* = 2.71 |
| … has a great value in our organization compared to other activities/goals. |  | Does not apply |  | Strongly applies |  | *n* = 7  *M* = 2.71 |
| **The intervention component “Extension of the kinesthetic lessons”** | | | | | | |
| … offers the apprentices a new capability to be physically active. |  | Does not apply |  | Strongly applies |  | *n* = 9  *M* = 3.00 |
| … contributes to the apprentices getting more physically active. |  | Does not apply |  | Strongly applies |  | *n* = 9  *M* = 2.56 |
| … is tailored to the needs and requirements of the apprentices. |  | Does not apply |  | Strongly applies |  | *n* = 9  *M* = 3.22 |
| … fits to the situation and the organizational conditions in our organization. |  | Does not apply |  | Strongly applies |  | *n* = 9  *M* = 2.89 |
| … has a great value in our organization compared to other activities/goals. |  | Does not apply |  | Strongly applies |  | *n* = 9  *M* = 3.11 |
| **The intervention component “Back-strengthening work / working posture”** | | | | | | |
| … offers the apprentices a new capability to be physically active. |  | Does not apply |  | Strongly applies |  | *n* = 8  *M* = 2.63 |
| … contributes to the apprentices getting more physically active. |  | Does not apply |  | Strongly applies |  | *n* = 8  *M* = 2.88 |
| … is tailored to the needs and requirements of the apprentices. |  | Does not apply |  | Strongly applies |  | *n* = 8  *M* = 3.25 |
| … fits to the situation and the organizational conditions in our organization. |  | Does not apply |  | Strongly applies |  | *n* = 8  *M* = 3.00 |
| … has a great value in our organization compared to other activities/goals. |  | Does not apply |  | Strongly applies |  | *n* = 8  *M* = 2.88 |
| **The intervention component “Motivation of care recipients”** | | | | | | |
| … offers the apprentices a new capability to be physically active. |  | Does not apply |  | Strongly applies |  | *n* = 8  *M* = 2.25 |
| … contributes to the apprentices getting more physically active. |  | Does not apply |  | Strongly applies |  | *n* = 8  *M* = 2.13 |
| … is tailored to the needs and requirements of the apprentices. |  | Does not apply |  | Strongly applies |  | *n* = 8  *M* = 2.50 |
| … fits to the situation and the organizational conditions in our organization. |  | Does not apply |  | Strongly applies |  | *n* = 8  *M* = 2.63 |
| … has a great value in our organization compared to other activities/goals. |  | Does not apply |  | Strongly applies |  | *n* = 8  *M* = 2.63 |
| **The intervention component “Information for practice trainers / head nurses”** | | | | | | |
| … offers the apprentices a new capability to be physically active. |  | Does not apply |  | Strongly applies |  | *n* = 6  *M* = 2.83 |
| … contributes to the apprentices getting more physically active. |  | Does not apply |  | Strongly applies |  | *n* = 6  *M* = 2.67 |
| … is tailored to the needs and requirements of the apprentices. |  | Does not apply |  | Strongly applies |  | *n* = 6  *M* = 2.83 |
| … fits to the situation and the organizational conditions in our organization. |  | Does not apply |  | Strongly applies |  | *n* = 6  *M* = 2.50 |
| … has a great value in our organization compared to other activities/goals. |  | Does not apply |  | Strongly applies |  | *n* = 6  *M* = 2.50 |
| **The intervention component “Cooperation with physiotherapy”** | | | | | | |
| … offers the apprentices a new capability to be physically active. |  | Does not apply |  | Strongly applies |  | *n* = 5  *M* = 2.60 |
| … contributes to the apprentices getting more physically active. |  | Does not apply |  | Strongly applies |  | *n* = 5  *M* = 2.80 |
| … is tailored to the needs and requirements of the apprentices. |  | Does not apply |  | Strongly applies |  | *n* = 5  *M* = 2.60 |
| … fits to the situation and the organizational conditions in our organization. |  | Does not apply |  | Strongly applies |  | *n* = 5  *M* = 2.40 |
| … has a great value in our organization compared to other activities/goals. |  | Does not apply |  | Strongly applies |  | *n* = 5  *M* = 2.40 |
| **The intervention component “Bonus system”** | | | | | | |
| … offers the apprentices a new capability to be physically active. |  | Does not apply |  | Strongly applies |  | *n* = 6  *M* = 2.83 |
| … contributes to the apprentices getting more physically active. |  | Does not apply |  | Strongly applies |  | *n* = 6  *M* = 3.00 |
| … is tailored to the needs and requirements of the apprentices. |  | Does not apply |  | Strongly applies |  | *n* = 6  *M* = 2.67 |
| … fits to the situation and the organizational conditions in our organization. |  | Does not apply |  | Strongly applies |  | *n* = 6  *M* = 2.67 |
| … has a great value in our organization compared to other activities/goals. |  | Does not apply |  | Strongly applies |  | *n* = 6  *M* = 2.33 |

Table 3: Appraisal of the intervention components in Setting C

| **The intervention component “Training module Physical Activity-related Health Competence“…** | | | | | | | | |
| --- | --- | --- | --- | --- | --- | --- | --- | --- |
| … offers the apprentices a new capability to be physically active. |  | Does not apply |  | Strongly applies | |  | | *n* = 3  *M* = 2.00 |
| … contributes to the apprentices getting more physically active. |  | Does not apply |  | Strongly applies | |  | | *n* = 3  *M* = 2.67 |
| … is tailored to the needs and requirements of the apprentices. |  | Does not apply |  | Strongly applies | |  | | *n* = 3  *M* = 3.67 |
| … fits to the situation and the organizational conditions in our organization. |  | Does not apply |  | Strongly applies | |  | | *n* = 3  *M* = 3.33 |
| … has a great value in our organization compared to other activities/goals. |  | Does not apply |  | Strongly applies | |  | | *n* = 3  *M* = 2.00 |
| **The intervention component “Tutoring system”…** | | | | | | | | |
| … offers the apprentices a new capability to be physically active. |  | Does not apply |  | Strongly applies | |  | | *n* = 5  *M* = 2.20 |
| … contributes to the apprentices getting more physically active. |  | Does not apply |  | Strongly applies | |  | | *n* = 5  *M* = 2.20 |
| … is tailored to the needs and requirements of the apprentices. |  | Does not apply |  | Strongly applies | |  | | *n* = 5  *M* = 2.40 |
| … fits to the situation and the organizational conditions in our organization. |  | Does not apply |  | Strongly applies | |  | | *n* = 5  *M* = 2.60 |
| … has a great value in our organization compared to other activities/goals. |  | Does not apply |  | Strongly applies | |  | | *n* = 5  *M* = 0.80 |
| **The intervention component “Information campaign for group leaders“…** | | | | | | | | |
| … offers the apprentices a new capability to be physically active. |  | Does not apply |  | Strongly applies | |  | | *n* = 3  *M* = 1.20 |
| … contributes to the apprentices getting more physically active. |  | Does not apply |  | Strongly applies | |  | | *n* = 3  *M* = 3.00 |
| … is tailored to the needs and requirements of the apprentices. |  | Does not apply |  | Strongly applies | |  | | *n* = 3  *M* = 2.67 |
| … fits to the situation and the organizational conditions in our organization. |  | Does not apply |  | Strongly applies | |  | | *n* = 3  *M* = 2.67 |
| … has a great value in our organization compared to other activities/goals. |  | Does not apply |  | Strongly applies | |  | | *n* = 3  *M* = 2.00 |
| **The intervention component “Opportunities for keeping fit“…** | | | | | | | | |
| … offers the apprentices a new capability to be physically active. |  | Does not apply |  | Strongly applies | |  | | *n* = 4  *M* = 2.50 |
| … contributes to the apprentices getting more physically active. |  | Does not apply |  | Strongly applies | |  | | *n* = 4  *M* = 2.25 |
| … is tailored to the needs and requirements of the apprentices. |  | Does not apply |  | Strongly applies | |  | | *n* = 4  *M* = 2.25 |
| … fits to the situation and the organizational conditions in our organization. |  | Does not apply |  | Strongly applies | |  | | *n* = 4  *M* = 2.75 |
| … has a great value in our organization compared to other activities/goals. |  | Does not apply |  | Strongly applies | |  | | *n* = 4  *M* = 0.50 |
| **The intervention component “Information strategy“…** | | | | | | | | |
| … offers the apprentices a new capability to be physically active. |  | Does not apply |  | Strongly applies |  | | *n* = 2  *M* = 2.50 | |
| … contributes to the apprentices getting more physically active. |  | Does not apply |  | Strongly applies |  | | *n* = 2  *M* = 1.00 | |
| … is tailored to the needs and requirements of the apprentices. |  | Does not apply |  | Strongly applies |  | | *n* = 2  *M* = 3.00 | |
| … fits to the situation and the organizational conditions in our organization. |  | Does not apply |  | Strongly applies |  | | *n* = 2  *M* = 1.50 | |
| … has a great value in our organization compared to other activities/goals. |  | Does not apply |  | Strongly applies |  | | *n* = 2  *M* = 0.50 | |
| **The intervention component “Coordininator / steering group“…** | | | | | | | | |
| … offers the apprentices a new capability to be physically active. |  | Does not apply |  | Strongly applies | |  | | *n* = 2  *M* = 2.50 |
| … contributes to the apprentices getting more physically active. |  | Does not apply |  | Strongly applies | |  | | *n* = 2  *M* = 1.00 |
| … is tailored to the needs and requirements of the apprentices. |  | Does not apply |  | Strongly applies | |  | | *n* = 2  *M* = 3.00 |
| … fits to the situation and the organizational conditions in our organization. |  | Does not apply |  | Strongly applies | |  | | *n* = 2  *M* = 1.50 |
| … has a great value in our organization compared to other activities/goals. |  | Does not apply |  | Strongly applies | |  | | *n* = 2  *M* = 0.50 |
