# Supplementary material for: Co-creating physical activity interventions: Findings from a multiple case study using mixed methods
Source: Front Public Health. 2022 Sep 21;10:975638. doi: 10.3389/fpubh.2022.975638 (PMC9534180; doi:10.3389/fpubh.2022.975638)
Supplement: Supplementary file 5 [file Table_5.docx]

Supplementary Material

# Additional File 5: Results of the organizational readiness assessment

Table 1: Results of the Kruskal-Wallis on the readiness for change (1 = disagree; 5 = agree)

|  | Total ORIC | | | Change Commitment | | | Change Efficacy | | |
| --- | --- | --- | --- | --- | --- | --- | --- | --- | --- |
|  | *n* | *M* | *SD* | *n* | *M* | *SD* | *n* | *M* | *SD* |
| Setting A | 16 | 3.77 | 1.04 | 16 | 3.76 | 1.11 | 16 | 3.77 | 1.01 |
| Setting B | 10 | 4.32 | 0.39 | 10 | 4.32 | 0.41 | 10 | 4.32 | 0.41 |
| Setting C | 9 | 3.54 | 0.54 | 9 | 3.64 | 0.69 | 9 | 3.44 | 0.60 |
| Kruskal-Wallis H-test | *H*(2) = 7.83, *p* = .020 * | | | *H*(2) = 4.61, *p* = .100 | | | *H*(2) = 9.00, *p* = .011 * | | |
| Post-hoc pairwise comparisons  (Dunn-Bonferroni tests) | Setting A vs. Setting B: *p* = .361  Setting B vs. Setting C: *p* = .015 *  Setting A vs. Setting C: *p* = .340 | | |  | | | Setting A vs. Setting B: *p* = .316  Setting B vs. Setting C: *p* = .008 *  Setting A vs. Setting C: *p* = .245 | | |
| *ORIC* Organizational Readiness for Implementing Change; * *p* < .05 | | | | | | | | | |
